# Supplementary material for: DMRforPairs: identifying Differentially Methylated Regions between unique samples using array based methylation profiles
Source: BMC Bioinformatics. 2014 May 15;15:141. doi: 10.1186/1471-2105-15-141 (PMC4046028; doi:10.1186/1471-2105-15-141)
Supplement: Additional file 2 — DMRforPairs output for the comparison of A431-MCF7 and NA17018-NA17105. Please start from the HTML files in each folder. Available via the BMC Bioinformatics website. [file 1471-2105-15-141-S2.zip › 1394847754114233_MOESM2_ESM/A431_MCF7/figures/27767.pdf]

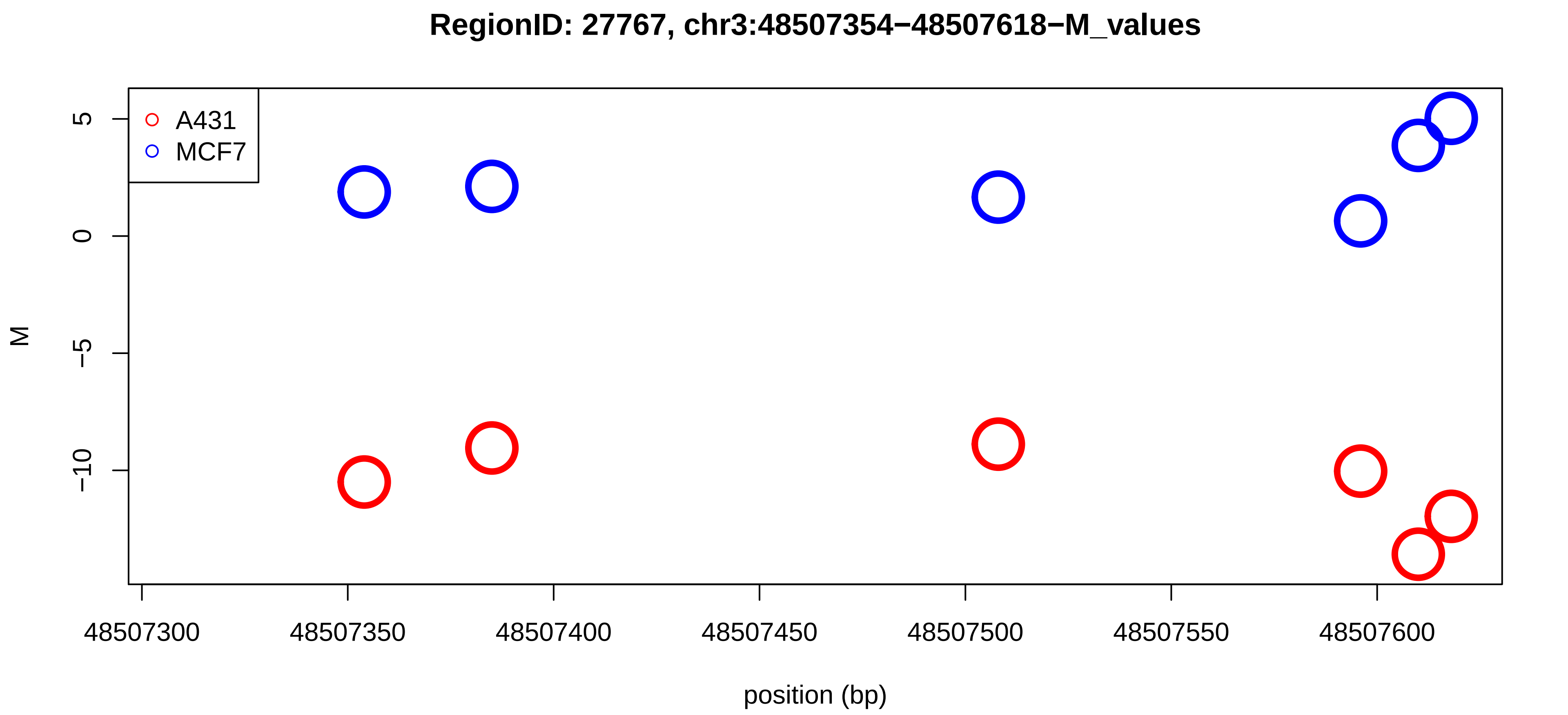
RegionID: 27767, chr3:48507354−48507618−M_values
A431
MCF7
M
5
0
−5
−10
48507300
48507350
48507400
48507450
48507500
48507550
48507600
position (bp)

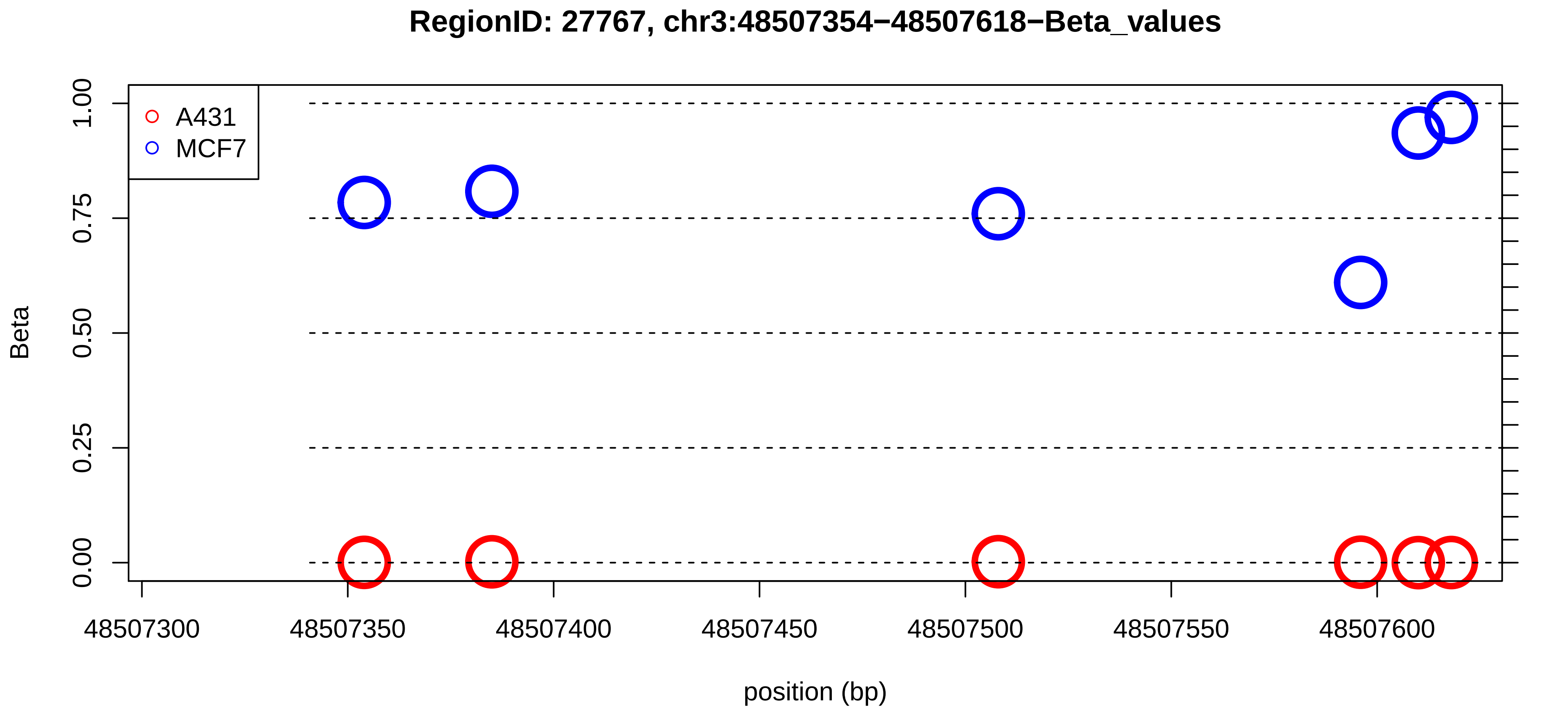
RegionID: 27767, chr3:48507354−48507618−Beta_values
A431
MCF7
Beta
1.00
0.75
0.50
0.25
0.00
48507300
48507350
48507400
48507450
48507500
48507550
48507600
position (bp)

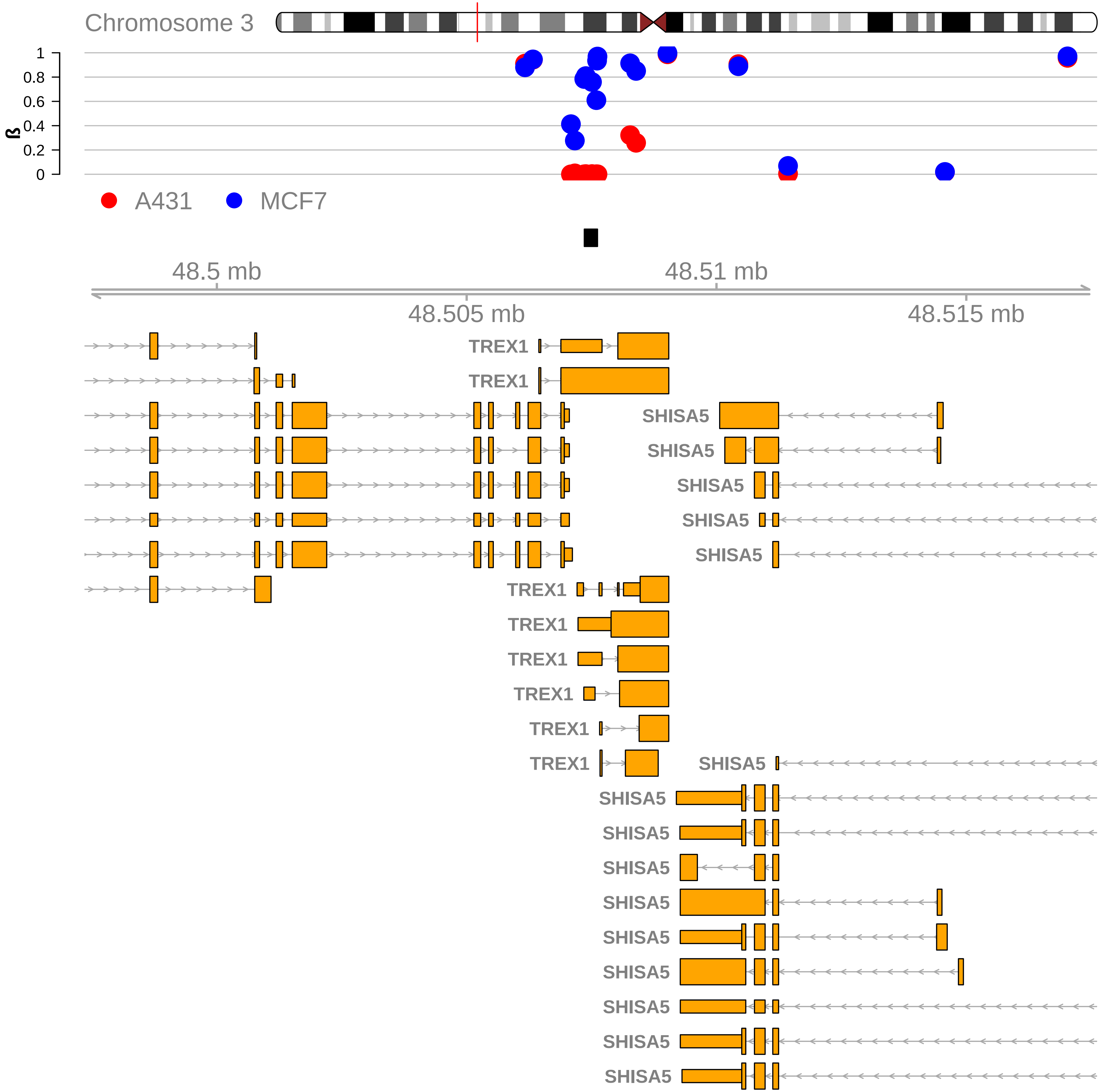
Chromosome 3
ß
1
0.8
0.6
0.4
0.2
0
A431
MCF7
48.5 mb
48.505 mb
48.51 mb
48.515 mb
TREX1
TREX1
SHISA5
SHISA5
SHISA5
SHISA5
SHISA5
TREX1
TREX1
TREX1
TREX1
TREX1
TREX1
SHISA5
SHISA5
SHISA5
SHISA5
SHISA5
SHISA5
SHISA5
SHISA5
SHISA5
SHISA5
